# Implementing a prediction driven framework for emergency department nurse staffing to optimize real time decisions

**DOI:** 10.1038/s44401-025-00019-2

**Published:** 2025-05-08

**Authors:** Yue Hu, Carri W. Chan, Jing Dong, Alice Kazekjian, Chayapol Ophaswongse, Gregory Sugalski, Joseph P. Underwood, Rimma Perotte

**Affiliations:** 1 Operations, Information & Technology, Stanford Graduate School of Business, Stanford, CA USA; 2 Decision, Risk, and Operations Division, Columbia Business School, New York, NY USA; 3 Department of Emergency Medicine, Hackensack University Medical Center, Hackensack, NJ USA; 4 Department of Emergency Medicine, Hackensack Meridian School of Medicine, Hackensack, NJ USA; 5 Department of Biomedical Informatics, Columbia University Irving Medical Center, New York, NY USA

**Keywords:** Health care economics, Health services, Information systems and information technology, Operational research, Engineering, Mathematics and computing

## Abstract

This study implemented and evaluated a prediction-driven nurse staffing framework in a large adult emergency department. The framework leveraged a two-stage prediction model that forecasted patient volume and guided staffing decisions. Using a pre-post study design, we compared patient throughput (measured by door-to-evaluation time, active treatment time, boarding time, length of stay, and left-without-being-seen rate) and cost outcomes (measured as hourly nurse staffing costs) before and after implementation. The model achieved an RMSE of 11.261 and MAPE of 13.414% at the base stage, and an RMSE of 9.973 and MAPE of 12.126% at the surge stage. The framework reduced hourly staffing costs by $162.04 without negatively affecting throughput. Reducing one nurse per hour from the recommended level increased wait times by two minutes, with an additional 2.3-min increase when staffing dropped below 20% of recommendations. These findings highlight the potential of prediction-driven staffing to reduce costs while maintaining patient throughput.

## Introduction

Emergency departments (EDs) are notorious for overcrowding and long wait times, which lead to delays in care, patient dissatisfaction, and increased costs^1,2^. One major driver of ED crowding is the lack of adequate clinical staffing to handle variations in patient volume^3^. Carefully aligning clinical staffing with patient demand is paramount to operating an efficient ED. Overstaffing can be prohibitively costly while understaffing leads to delays in care, increased walkout rates, and staff burnout^4,5^.

Nurse staffing decisions in EDs typically follow a two-stage process, consisting of a “base” stage and a “surge” stage. In the base stage, initial schedules are set weeks in advance based on average patient volume and staffing ratios^6^. However, unexpected surges in patient volume often necessitate additional resources on short notice at the surge stage^7,8^. To accommodate these fluctuations, “surge” nurses are usually offered incentive pay, which is substantially higher than the standard compensation rate.

Numerous studies have developed predictive models for ED demand and occupancy using techniques such as time-series models (sometimes incorporating exogenous features)^9–11^, regression^12–14^, and other machine learning (ML) approaches^15,16^. While these predictive insights could inform staffing decisions, most existing research focuses on forecasting demand at a single fixed time point. This misalignment with the two-stage staffing process makes it unclear how to effectively incorporate predictive models into real-world staffing decisions.

Recently, Hu et al.^17^ introduced a two-stage prediction model designed to match the two staffing decision points. Their approach utilizes a base-stage prediction model that relies on relatively coarse information, while the surge-stage prediction model incorporates more real-time data as the shift approaches. A subsequent study^18^ expanded on this framework by integrating predictive analytics with prescriptive staffing recommendations through mathematical modeling and statistical analysis. However, this framework remained theoretical and had not yet been deployed or evaluated in a real-world ED setting. Our study aims to bridge this gap by implementing and assessing the impact of this prediction-driven nurse staffing framework in a large, high-volume adult ED.

The prediction-driven staffing framework developed by Hu et al.^17,18^ utilizes a two-stage prediction model for patient demand. In the base stage, which takes place several weeks in advance, nurse demand is predicted based on limited information (e.g., day of the week, time of day). The surge stage, occurring one day before the shift, utilizes richer real-time data (e.g., ED census, weather forecast) to refine the nurse demand prediction. If the surge stage predicts that more nurses are required than previously indicated in the base stage, it informs nurse leadership of how many extra (surge) nurses should be called in.

Our study built upon the work of Hu et al.^17,18^ by pioneering the implementation of this methodological framework within a real ED setting. Utilizing Google Apps Script, we developed a digital staffing tool and piloted the prediction-driven staffing framework for over four months. Through this pilot study, we gained valuable insights into the construction of a robust implementation infrastructure in ED nurse staffing practice. More importantly, we evaluated the operational efficiency and access to care gained from this prediction-driven nurse staffing framework.

## Results

We begin by presenting the findings from a four-month pilot implementation of the prediction-driven staffing framework. A detailed description of the study design, implementation setting, and evaluation metrics is provided in the Methods section below.

### Summary statistics across the control and experimental stages

Descriptive statistics comparing the control and experiment stages are presented in Table 1. The average number of patients per day was 244.77 in the control stage and 249.03 in the experiment stage (*p* = 0.287). Patient demographics, including age (52.27 vs. 52.19, *p* = 0.756) and gender distribution (54.55% vs. 55.07% female, *p* = 0.993), were similar across both periods. Staffing patterns showed notable differences. The average number of surge nursing hours per hour decreased from 10.01 in the control stage to 7.51 in the experiment stage (*p* < 0.001), while the base staffing hours remained consistent (11.1 vs. 11.09, *p* = 0.218). Key patient throughput metrics remained stable between the two periods. Average treatment time (4.75 vs. 4.93 h, *p* = 0.197), boarding time (4.7 vs. 4.25 h, *p* = 0.127), length of stay (LOS) (9.55 vs. 9.14 h, *p* = 0.220), and leaving without being seen (LWBS) rate (0.10% vs. 0.11%, *p* = 0.910) showed no statistically significant differences. However, the average waiting time increased slightly from 0.49 hours in the control stage to 0.55 h in the experiment stage (*p* = 0.019).

**Table 1:** Summary statistics compared between the control and experiment stages

	Control	Experiment	*P*-value (KS test)
Average # patients per day	244.77	249.03	0.287
Average age	52.27	52.19	0.756
Gender			
Female	54.55%	55.07%	0.993
Male	45.45%	44.93%	
Arrival means			
Ambulance	26.40%	25.60%	0.804
Walk-in	73.60%	74.40%	
Acuity level			
ESI-1	7.63%	7.34%	0.124
ESI-2	25.88%	25.02%	
ESI-3	53.98%	53.76%	
ESI-4	11.54%	12.77%	
ESI-5	0.97%	1.11%	
Insurance type			
Private	39.48%	38.39%	0.000
Medicare	25.71%	24.64%	
Medicaid	21.93%	21.86%	
Self-Pay	11.50%	13.89%	
Charity	1.38%	1.22%	
Average # base nursing hours per hour	11.1	11.09	0.218
Average # surge nursing hours per hour	10.01	7.51	0.000
Average waiting time (hours)	0.49	0.55	0.019
Average treatment time (hours)	4.75	4.93	0.197
Average boarding time for admitted patients (hours)	4.7	4.25	0.127
Average LOS (hours)	9.55	9.14	0.220
Percentage of LWBS	0.10%	0.11%	0.910

Nurse staffing composition by experience and shift types is presented in Table 2. During the experiment stage, the proportion of novice nurses (<1 year of experience) increased from 19 to 29, while the number of expert nurses (>5 years of experience) declined from 18 to 13 compared to the control stage. In addition, the proportion of regular/incentive nurse hours increased from 69% to 81%, while reliance on travel nurses decreased from 31% to 19%.

**Table 2:** Nurse staffing composition by experience and shift type

Category	Details	Control	Experiment
Skill level		Count	
Novice	<1 year of experience	28	29
Beginner	up to 2 years of experience	5	3
Competent	up to 3 years of experience	3	4
Proficient	up to 5 years of experience	6	4
Expert	>5 years of experience	18	13
**Total nurses**		**60**	**53**
**Shift type**		**Percentage**	
Regular/incentive	% of total nursing hours	73%	81%
Travelers	% of total nursing hours	27%	19%

### Time-series comparison of ED census and staffing levels

**Fig. 1:**
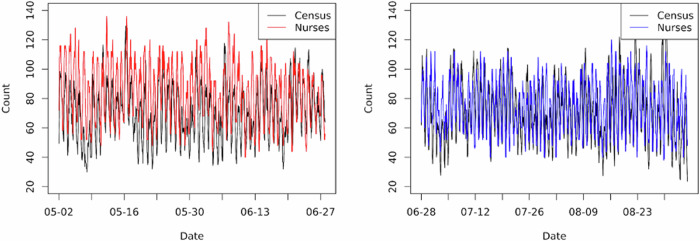
Time series comparison of ED Census and staffing levels.

Figure 1 displays time-series plots comparing hourly ED census and staffing levels during the control (left) and experiment (right) stages. The mean squared differences were 491.39 for control and 323.97 for experiment. The prediction-driven staffing reduced the mean squared difference by 34.07%, highlighting a more precise alignment between workload and staffing.

### Tradeoff curves

**Fig. 2:**
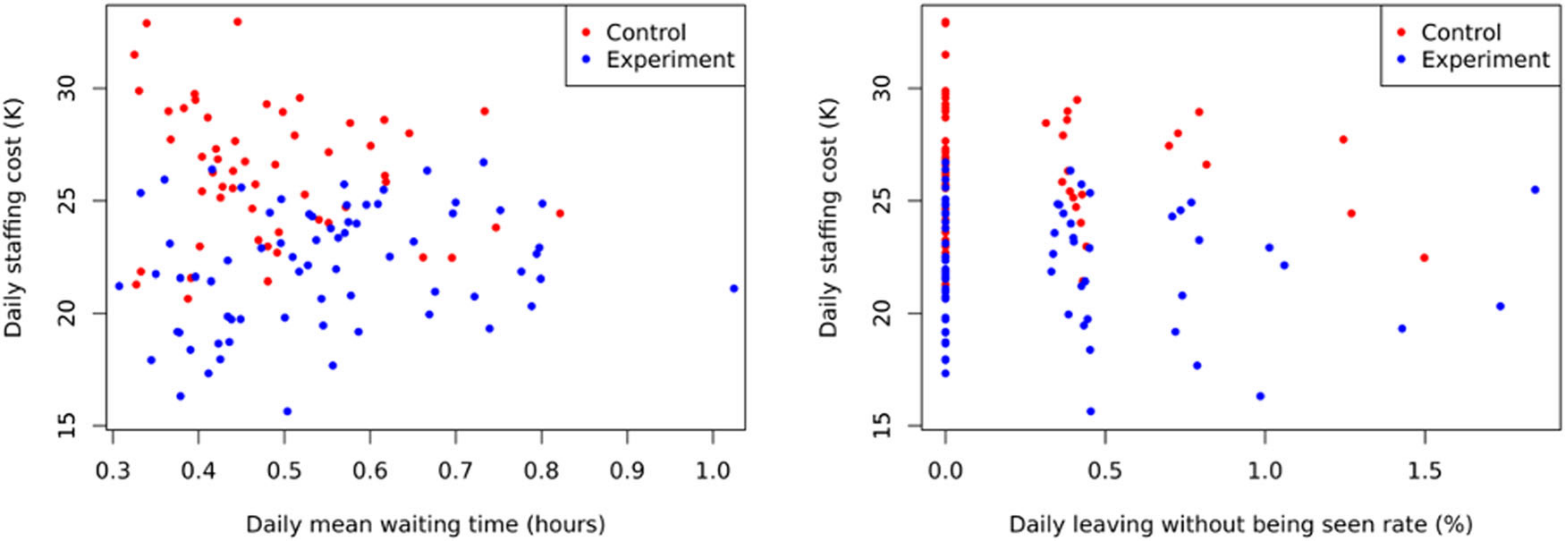
Tradeoff curves balancing quality and efficiency.

The tradeoff curves in Fig. 2 demonstrate how the traditional staffing practice and the prediction-driven staffing framework balanced staffing costs and access-to-care metrics. Each data point in the figure represented one day of operation during the control (red) and the experiment (blue) stages. The left subfigure depicted staffing costs versus average waiting times, while the right subfigure showed staffing costs versus LWBS rates. We observed that the tradeoff curve of the experiment stage lay below that of the control stage, which suggested that the prediction-driven staffing framework, by analytically optimizing resource allocation, achieved a more adept balance between staffing costs and service quality, i.e., for the same access-to-care target, the new staffing practice could achieve it with a lower staffing cost.

### Differences in staffing efficiency across the control and experimental stages

The linear regression outputs are summarized in Table 3. The regression achieved an adjusted R-squared value of 0.734. The coefficient for the indicator of the experiment stage was statistically significant with *p*-value less than 0.001. This indicator had a fitted coefficient of −162.044, suggesting that, with all else equal, the experiment stage reduced hourly staffing costs by $162.044 compared to the control stage. In other words, the prediction-driven staffing framework achieved comparable access-to-care targets with a lower staffing cost.

**Table 3:** Linear regression model for staffing efficiency (12-h window)

Covariate	Coefficient	Standard error	*P*-value
Intercept	849.620	116.281	<0.001
Monday 7 am – 7 pm	−45.954	41.957	0.275
Monday 7 pm – 7 am (next day)	−226.637	76.254	0.003
Tuesday 7 am – 7 pm	42.865	39.151	0.275
Tuesday 7 pm – 7 am (next day)	−164.965	72.583	0.024
Wednesday 7 am – 7 pm	75.723	38.522	0.051
Wednesday 7 pm – 7 am (next day)	−195.187	72.370	0.008
Thursday 7 am – 7 pm	92.157	38.567	0.018
Thursday 7 pm – 7 am (next day)	−160.741	71.087	0.025
Friday 7 pm – 7 am (next day)	−273.024	71.522	<0.001
Saturday 7 am – 7 pm	−65.971	41.772	0.116
Saturday 7 pm – 7 am (next day)	−235.034	68.682	<0.001
Sunday 7 am – 7 pm	−171.030	45.263	<0.001
Sunday 7 pm – 7 am (next day)	−276.879	70.112	<0.001
Average arrival count per hour	17.039	7.825	0.030
Number of waiting patients at the beginning of the period	0.243	2.175	0.911
Number of patients in treatment at the beginning of the period	3.146	1.002	0.002
Number of boarding patients at the beginning of the period	0.337	0.800	0.674
Average patient waiting time (minutes)	−3.441	0.895	<0.001
Average patient LOS (minutes)	0.252	0.093	0.007
LWBS rate (%)	−27.964	11.136	0.013
Experiment stage indicator	−162.044	15.339	<0.001

Additional covariates showed expected relationships with staffing costs. Average patient arrival count per hour (coefficient = 17.039, *p* = 0.030), number of patients in treatment at the beginning of the period (coefficient = 3.146, *p* = 0.002), and average LOS for newly arriving patients (coefficient = 0.252, *p* = 0.007) were positively associated with staffing costs. Patient waiting time (coefficient = −3.441, *p* < 0.001) and LWBS rate (coefficient = −27.964, *p* = 0.013) were negatively correlated with staffing costs. This suggested that reducing the average patient waiting time by 1 min corresponded to an increase of $3.441 in hourly staffing costs, with all other factors held constant. Similarly, reducing the LWBS rate by 1% translated to an increase of $27.964 in hourly staffing costs.

Regression analyses were also conducted for 4-h, 2-h, and 1-h intervals, detailed in Supplementary Note 2. The results remained consistent across different time window durations. Importantly, the coefficients related to the experiment-stage indicator were consistent, indicating similar cost-saving estimates during the experimental stage.

### Impact of and rationale for not following the staffing levels recommended by the system

**Fig. 3:**
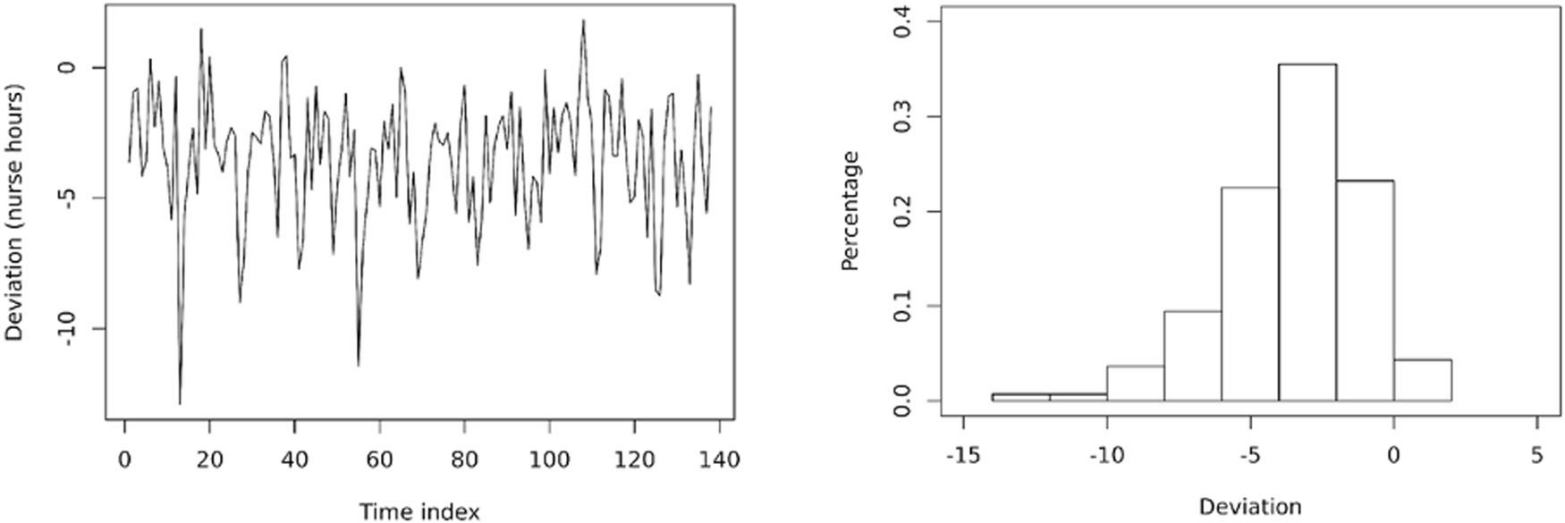
Time-series plot and histogram of staffing deviations.

**Fig. 4:**
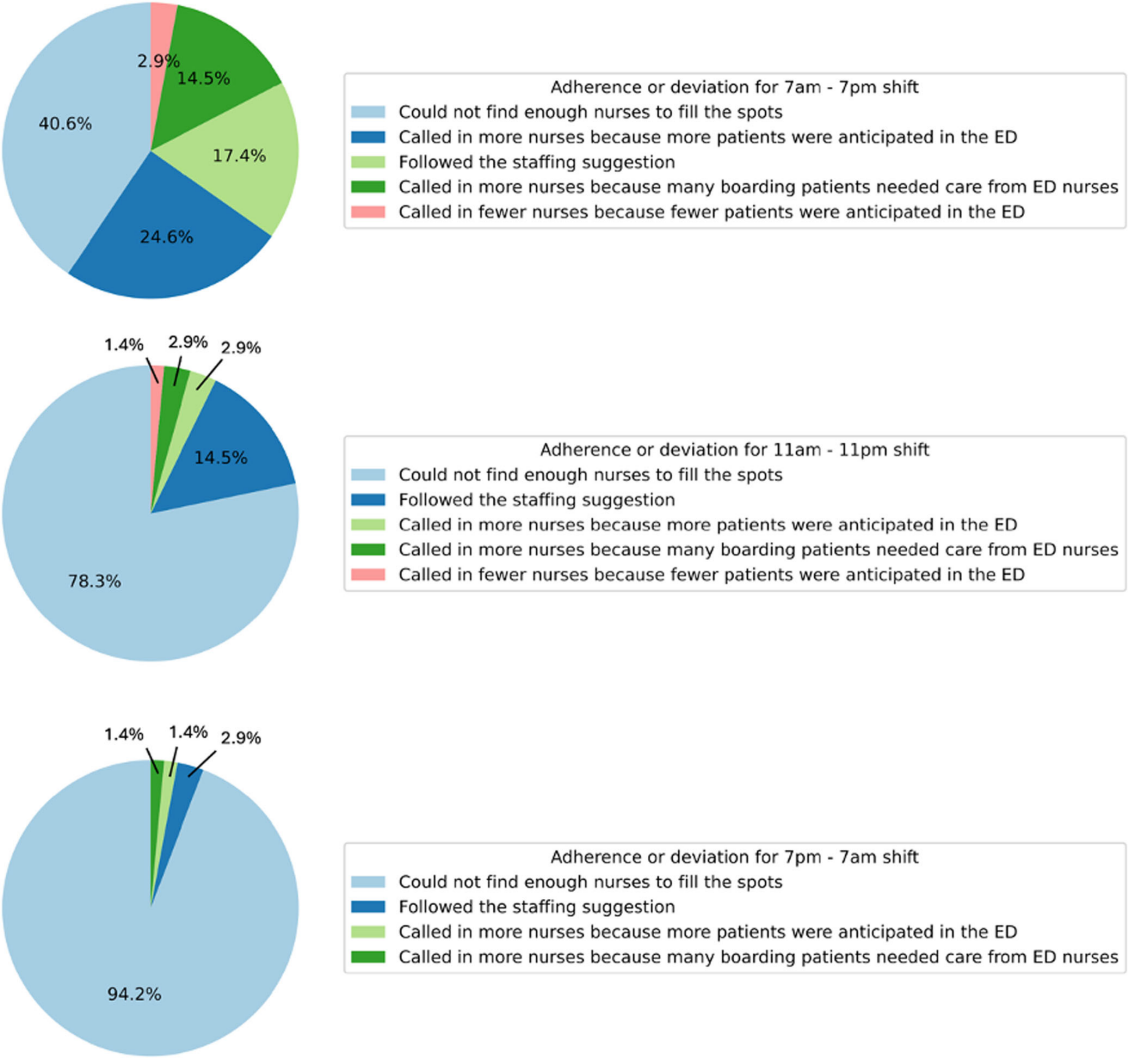
Reported adherence or deviation distribution (100% completion rate).

In our analysis of staffing discrepancies, Fig. 3 visualizes deviations across 69 days, with a mean deviation of −3.5 nursing hours per hour and a standard deviation of 2.5 nursing hours per hour, primarily indicating an understaffing trend. The negative nursing hours of 3.5 per hour can be interpreted as missing 3.5 nurses on the floor at any given time on average. In the pie chart shown in Fig. 4, we summarized the reasons for adherence and deviations reported by nursing leadership using the email links. The top three frequently reported patterns were: 1) “Could not find enough nurses to fill the spots,” - classified as a deviation because of limited staffing ability, 2) “Followed the staffing suggestion,” - classified as adherence, and 3) “Called in more nurses because more patients were anticipated in the ED.” - classified as a deviation because of the department being busier than the recommendation predicted. Among the 7 am – 7 pm shifts, for example, adherence was reported 17.4% of the time, while “Could not find enough nurses to fill the spots” was reported 40.6% of the time.

To assess the impact of staffing deviations on patient waiting times, Table 4 presents results from a linear regression analysis, with an adjusted R-squared value of 0.078. Two crucial staffing deviation variables—the deviation level and the indicator for understaffing by more than 20%—were statistically significant at the 5% significance level. The regression results suggested that a deficit of 1 nurse per hour from the recommended level led to a 2-min increase in each patient’s waiting time, with all other variables constant. Furthermore, if the staffing level fell below the recommendation by more than 20%, the patient’s waiting time increased by 2.3 min.

**Table 4:** Linear regression model for deviation analysis (patient level)

Covariate	Coefficient	Standard error	*P*-value
Intercept	−52.890	17.214	0.002
Monday 7 am – 11 am	−10.402	3.742	0.005
Monday 11 am – 7 pm	−61.326	16.484	<0.001
Monday 7 pm – 11 pm	−15.811	13.845	0.253
Monday 11 pm – 7 am (next day)	−2.915	2.881	0.312
Tuesday 7 am – 11 am	−5.760	2.519	0.022
Tuesday 11 am – 7 pm	−51.295	12.118	<0.001
Tuesday 7 pm – 11 pm	−10.522	10.304	0.307
Tuesday 11 pm – 7 am (next day)	2.936	2.874	0.307
Wednesday 7 am – 11 am	−4.643	2.327	0.046
Wednesday 11 am – 7 pm	−47.312	11.120	<0.001
Wednesday 7 pm – 11 pm	−11.151	10.203	0.274
Wednesday 11 pm – 7 am (next day)	−4.829	2.922	0.098
Thursday 7 am – 11 am	−4.841	2.560	0.059
Thursday 11 am – 7 pm	−48.972	10.978	<0.001
Thursday 7 pm – 11 pm	−16.595	9.254	0.073
Thursday 11 pm – 7 am (next day)	−3.263	2.755	0.236
Friday 11 am – 7 pm	−43.843	9.268	<0.001
Friday 7 pm – 11 pm	−10.132	9.303	0.276
Friday 11 pm – 7 am (next day)	−4.141	2.711	0.127
Saturday 7 am – 11 am	1.048	2.489	0.674
Saturday 11 am – 7 pm	−33.494	7.390	<0.001
Saturday 7 pm – 11 pm	−7.911	6.601	0.231
Saturday 11 pm – 7 am (next day)	4.720	2.775	0.089
Sunday 7 am – 11 am	−2.156	2.566	0.401
Sunday 11 am – 7 pm	−41.918	10.207	<0.001
Sunday 7 pm – 11 pm	−12.778	9.289	0.169
Sunday 11 pm – 7 am (next day)	−1.165	2.845	0.682
Insurance type – Medicaid	−3.601	2.379	0.130
Insurance type – Medicare	−4.625	2.443	0.058
Insurance type – private	−5.090	2.347	0.030
Insurance type – self-pay	−5.350	2.425	0.027
Acuity level 2	12.793	1.110	<0.001
Acuity level 3	16.655	1.083	<0.001
Acuity level 4	7.103	1.294	<0.001
Acuity level 5	3.684	2.775	0.184
Age group (40, 60)	4.235	0.670	<0.001
Age group (60, 80)	6.911	0.846	<0.001
Age group ≥80	4.549	1.231	<0.001
Arrival means – walk-in	8.808	0.666	<0.001
Average arrival count per hour	0.393	0.027	<0.001
Number of waiting patients at the beginning of the hour	1.178	0.077	<0.001
Number of patients in treatment at the beginning of the hour	0.269	0.035	<0.001
Number of boarding patients at the beginning of the hour	0.112	0.028	<0.001
Number of base nurses staffed for the hour	1.400	0.999	0.161
Number of surge nurses staffed for the hour	1.173	0.994	0.238
Indicator of overstaffing by 20% compared to the recommendation for the hour	1.154	2.023	0.568
Indicator of understaffing by 20% compared to the recommendation for the hour	2.346	1.024	0.022
Actual staffing level minus recommended staffing level for the hour	−2.022	0.995	0.042

## Discussion

The prediction-driven staffing framework navigates a delicate tradeoff between prediction accuracy and staffing costs. The continuous integration of real-time information can improve the accuracy of demand predictions over time. However, this advantage needs to be weighed against the fact that a significant portion of staffing decisions (e.g., the base staff) has to be made well in advance (e.g., four to six weeks prior in our hospital) when demand forecasts are less precise. As the shift starts nearer, the updated surge-stage prediction model supplies more refined demand information, allowing for the strategic deployment of additional surge staff. This, however, requires extra incentive pay for staff availability and responsiveness.

Our pilot study implemented the prediction-driven staffing framework in a real hospital setting and demonstrated the effectiveness of this innovative approach. The base-stage linear regression model achieved a test RMSE of 11.261 and a test MAPE of 13.414%, while the surge-stage linear regression model improved performance to a test RMSE of 9.973 and a test MAPE of 12.126%. With more real-time information, the surge-stage prediction model reduced RMSE by 11.438% and MAPE by 9.062% compared to the base-stage prediction model. To keep the focus of this paper on the pilot evaluation, we relegate details on developing the two-stage prediction models to Supplementary Note 1. To further contextualize our model’s performance, we compared our results with overlapping models from Hu et al.^17^, who developed them to forecast arrivals in another ED setting. Their study reported that a linear regression model without real-time information (similar to our base-stage model) achieved a test RMSE of 16.425 and a test MAPE of 9.627%, while an enhanced linear regression model incorporating real-time information (similar to our surge-stage model) improved performance to a test RMSE of 15.336 and a test MAPE of 9.109%. In comparison, our models achieved notably improved RMSE, while MAPE performance remained comparable.

Applying the two-stage staffing rule translated the improved prediction accuracy into enhanced staffing efficiency. Our findings revealed reduced hourly staffing costs by $162.044 while guaranteeing similar access-to-care metrics, including the average patient waiting time and LWBS rates. This implied an annual saving in total nurse staffing costs of $1.4 million, based on a rate of $162.044 per hour, operating 24 h a day, 365 days a year, a significant amount given that EDs often operate on tight budgets. Notably, this improvement was achieved despite a decline in total nursing staff from 60 to 53 and a reduction in the number of expert nurses from 18 to 13 in the experiment stage compared to the control stage. Additionally, the greater reliance on core staff and reduced dependence on travel nurses, reflected in the increase of regular/incentive nurse hours from 73% to 81% and the decline in travel nurse hours from 27% to 19%, suggested a more sustainable and stable workforce model.

The observed improvement in staffing efficiency could be attributed to the framework’s ability to predict and adjust nurse allocation more efficiently. By optimizing surge staffing in response to predicted patient demand, the system reduced unnecessary labor costs while maintaining overall service levels. That said, although most patient throughput metrics remained stable, the slight increase in patient waiting time suggested that even small deviations from recommended staffing levels could impact patient flow. Our results showed that reducing one nurse per hour from the recommendation led to a 2-min increase in waiting time, while staffing deviations exceeding 20% of the recommendation resulted in an additional 2.3-min delay. This underscored the importance of strict adherence to staffing recommendations to maintain optimal ED performance.

Beyond the observed efficiency gains in our pilot study, the prediction-driven staffing framework holds broader implications, particularly its adaptability to other hospital units and health systems. However, ED leaders contemplating a similar approach should note two key considerations. First, our deviation analysis highlighted challenges faced by nursing leadership in following staffing recommendations, mainly due to the difficulties in securing enough nurses on short notice. In our study setting, the incentive pay remained fixed regardless of the notice period. While this structure streamlined administrative processes, it may have limited the effectiveness of attracting additional nurses when urgent staffing needs arose. To address real-time staffing shortages, ED management could consider adjusting incentive pay to offer higher compensation for nurses called in on shorter notice. Alternatively, collaboration with nearby nursing agencies offering short-term locum nurses could help address staffing gaps, though feasibility may vary by hospital, and concerns regarding care quality remain^19–21^. Second, while our hospital sets surge predictions and thus surges staffing decisions one day in advance, shorter lead times could improve prediction accuracy. Striving to shorten the lead times could enhance the performance of the prediction-driven staffing framework. However, such an enhancement has implications for staffing-related expenses, depending on the float pool structure and the relationship between lead time and hourly rate.

The implementation of a prediction-driven staffing framework has the potential to impact nurse burnout and job satisfaction in both positive and negative ways. On the positive side, the framework could reduce nurse burnout by ensuring enough staff to meet patient demand, thereby reducing workload and stress levels. Additionally, the framework could improve job satisfaction by giving nurses more control over their schedules and allowing them to earn extra income through surge pay. However, it is important to note that the framework could also negatively impact nurse burnout and job satisfaction. For example, the framework could lead to increased stress and anxiety among nurses if they are constantly called in to work on short notice. Additionally, the framework could create a sense of unfairness among nurses if some nurses are consistently called in to work on surge shifts while others are not. Therefore, it is essential to carefully consider the potential impact of the prediction-driven staffing framework on nurse burnout and job satisfaction before implementing it in a real-world setting. Strategies to mitigate the framework’s negative impacts, such as ensuring that surge calls and pay are distributed fairly, must also be developed.

Similarly, implementing a prediction-driven staffing framework could impact patient outcomes in both positive and negative ways. On the positive side, the framework could improve patient outcomes by ensuring enough staff to meet patient demand, thereby reducing wait times and improving the quality of care. Additionally, the framework could reduce the risk of medical errors by ensuring that nurses’ workloads are properly managed. However, it is important to note that the framework could also negatively impact patient outcomes. For example, it could lead to increased patient wait times if there are not enough surge nurses available to meet demand. Additionally, the framework could increase the risk of medical errors if nurses are constantly called in to work on short notice and do not have adequate time to prepare. Therefore, it is essential to consider the potential impact of the prediction-driven staffing framework on patient outcomes before implementing it in the real world. It is also important to develop strategies to mitigate the framework’s negative impacts, such as ensuring that there are enough surge nurses available to meet demand and providing nurses with adequate support and resources.

In exploring potential improvements to our framework, we explored the use of Extreme Gradient Boosting (XGBoost) and Artificial Neural Networks (ANN) for surge-stage demand prediction to assess whether more advanced machine learning models could improve forecasting accuracy. However, this exploration was limited to a basic implementation, and more refined approaches may yield better results. These models were evaluated against our regression-based models solely for prediction accuracy and were not employed in the pilot for staffing decisions. While both XGBoost and ANN outperformed the base-stage prediction model, neither achieved better accuracy than the surge-stage linear regression model (Supplementary Note 1). This finding aligns with prior research in another ED setting by Hu et al.^17^, where XGBoost did not provide meaningful improvements over simpler statistical models. One possible reason is that more complex models like XGBoost and ANN, which typically excel in highly nonlinear and high-dimensional settings, may have overfit to the training data and struggled to generalize. Additionally, given the limited number of predictive features available, the added complexity of ML models may not offer significant advantages over a well-regularized linear regression approach in this context. Future research could explore alternative ML models, more extensive hyperparameter tuning, or enhanced feature engineering to determine whether AI-based approaches can yield greater improvements in ED demand forecasting.

While our findings demonstrate the promise of prediction-driven staffing, certain limitations should be acknowledged to guide future work.

This study used a pre-post design within a single ED, without a concurrent control group (i.e., a comparable ED without the intervention). While this allowed for a direct within-site comparison, it could be subject to temporal confounders. To mitigate this concern, we controlled for time-varying factors in our regression analysis. A concurrent control could have strengthened causal inference, but identifying a comparable ED with similar operational constraints was not feasible. Additionally, a Difference-in-Differences (DiD) model using historical data with parallel pre-trends could have provided a stronger causal estimate by accounting for system-wide fluctuations. However, the COVID-19 pandemic disrupted healthcare routines, making it difficult to establish parallel pre-trends between our study period and historical years. Future research should explore multi-site evaluations or DiD analysis, ideally using post-pandemic historical data, to further validate the model’s impact.

Our pilot was conducted from May to September, which did not capture the full annual variability in ED demand. While seasonal fluctuations could impact patient arrivals, the control and experiment periods occurred within a similar time window, helping to mitigate seasonal effects. Additionally, we controlled for seasonal trends in our regression analysis. However, future studies should evaluate model performance over a full year to assess potential seasonal influences more comprehensively.

Lastly, while our study demonstrated the potential cost savings of the prediction-driven staffing framework, it is important to acknowledge that a more nuanced approach to base staffing could be developed. The notable seven-day cycle in surge staffing suggests that there may be regular, predictable fluctuations in patient volume that could be addressed by adjusting the baseline staffing levels. This insight highlights the importance of evaluating and optimizing baseline and surge staffing to ensure efficient and effective nurse allocation in the ED.

In conclusion, this study provides valuable insights into the potential benefits and challenges of implementing a prediction-driven staffing framework in a real-world ED setting. While further research is needed to explore the full potential of this approach, it offers a promising avenue for improving cost-effectiveness while maintaining high-quality patient care. It is also essential to consider the potential impact of the framework on nurse burnout, job satisfaction, and patient outcomes and to develop strategies to mitigate any adverse effects. The framework could be further enhanced by integrating it with other technologies, such as AI and ML, to improve its accuracy and efficiency.

## Methods

### Study setting and objective

The prediction-driven nurse staffing framework was implemented in an adult ED at a large academic medical center with approximately 90,000 annual patient encounters. In our study, “nurses” refer exclusively to registered nurses (RNs), and they were the primary clinical staff subject to staffing decisions in the prediction-driven framework. To evaluate the framework performance, we divided the time horizon into three stages: trial, control, and experiment. We considered outcome variables that reflect cost, operational efficiency, and quality of care, as commonly studied in the literature on ED performance^22–24^. These include hourly staffing costs, as well as patient throughput measured by average door-to-evaluation time, active treatment time, boarding time, LOS, and LWBS rates; see the Pilot Evaluation Metrics subsection for details. The Columbia University Institutional Review Board approved this study: Protocol AAAT6452.

The trial stage spanned 21 days, from April 11, 2022, to May 1, 2022, during which the prediction-driven staffing system underwent testing. Based on discussions with our ED leadership, a three-week trial period was deemed sufficient for staff to gain familiarity with the system while ensuring timely feedback for refinement before full implementation. Each day during the trial stage, between 11 am and noon, the ED nursing leadership received a system-generated email similar to those sent during the experiment stage. However, compliance with the new system’s recommendations was optional, and the primary objective was to familiarize the nursing leadership with the system. Feedback was actively sought from the nursing leadership towards the conclusion of the trial period to enhance the user interface of this new nurse staffing recommendation system.

The control stage lasted 57 days, from May 2, 2022, to June 27, 2022. The primary objective was to collect data to assess the performance of the existing nurse staffing approach. Throughout this phase, the ED nursing leadership maintained the existing staffing protocols, and the email notifications from the new system were suspended to mitigate potential bias. During this stage, the base staffing template (Table 5) for the experiment stage (as prescribed by the two-stage prediction-driven staffing framework) was finalized and implemented. The base staffing decisions were made six weeks before the experiment stage. Note that the base staffing model was part of the two-stage prediction-driven staffing framework and had not been previously employed by the ED.

**Table 5:** Recommended base staffing levels during the experiment stage

	7 AM – 7 PM	11 AM – 11 PM	7 PM – 7 AM
Sunday	17	10	15
Monday	20	12	17
Tuesday	18	10	16
Wednesday	16	10	16
Thursday	18	9	16
Friday	17	9	17
Saturday	17	7	16

**Fig. 5:**
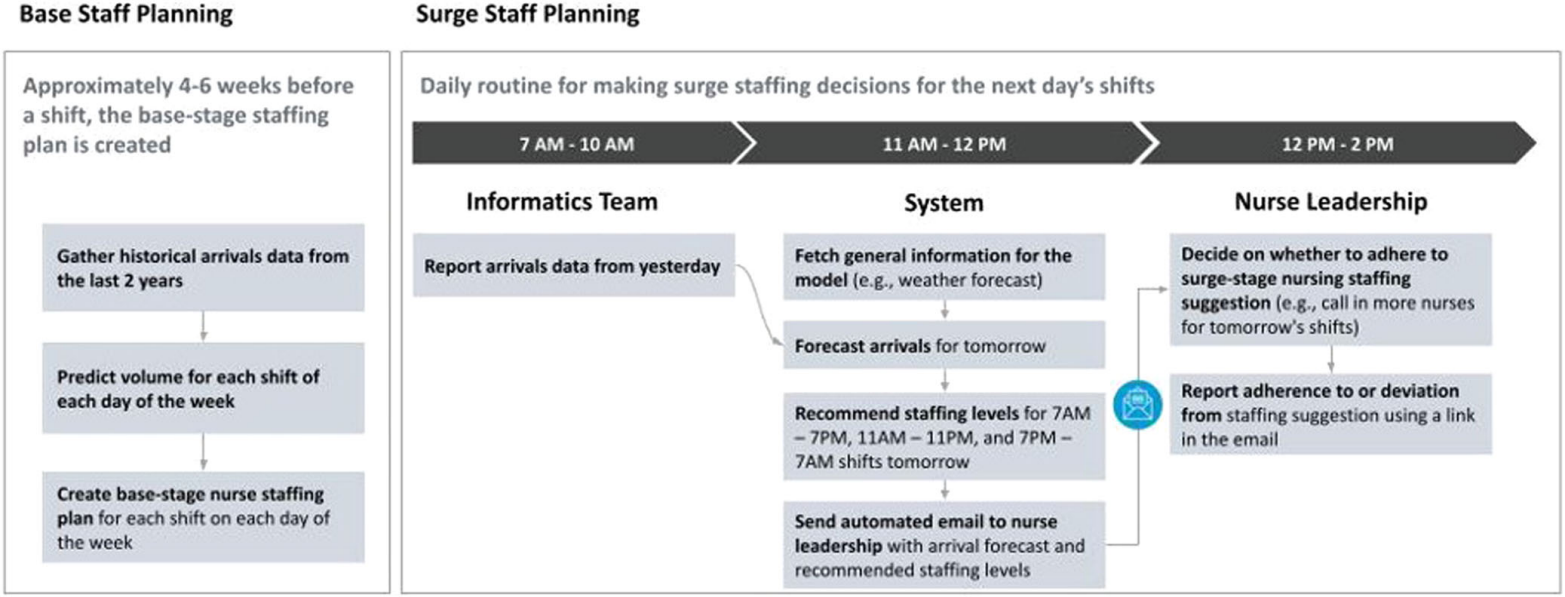
Staffing planning and daily routines during the experiment stage.

**Fig. 6:**
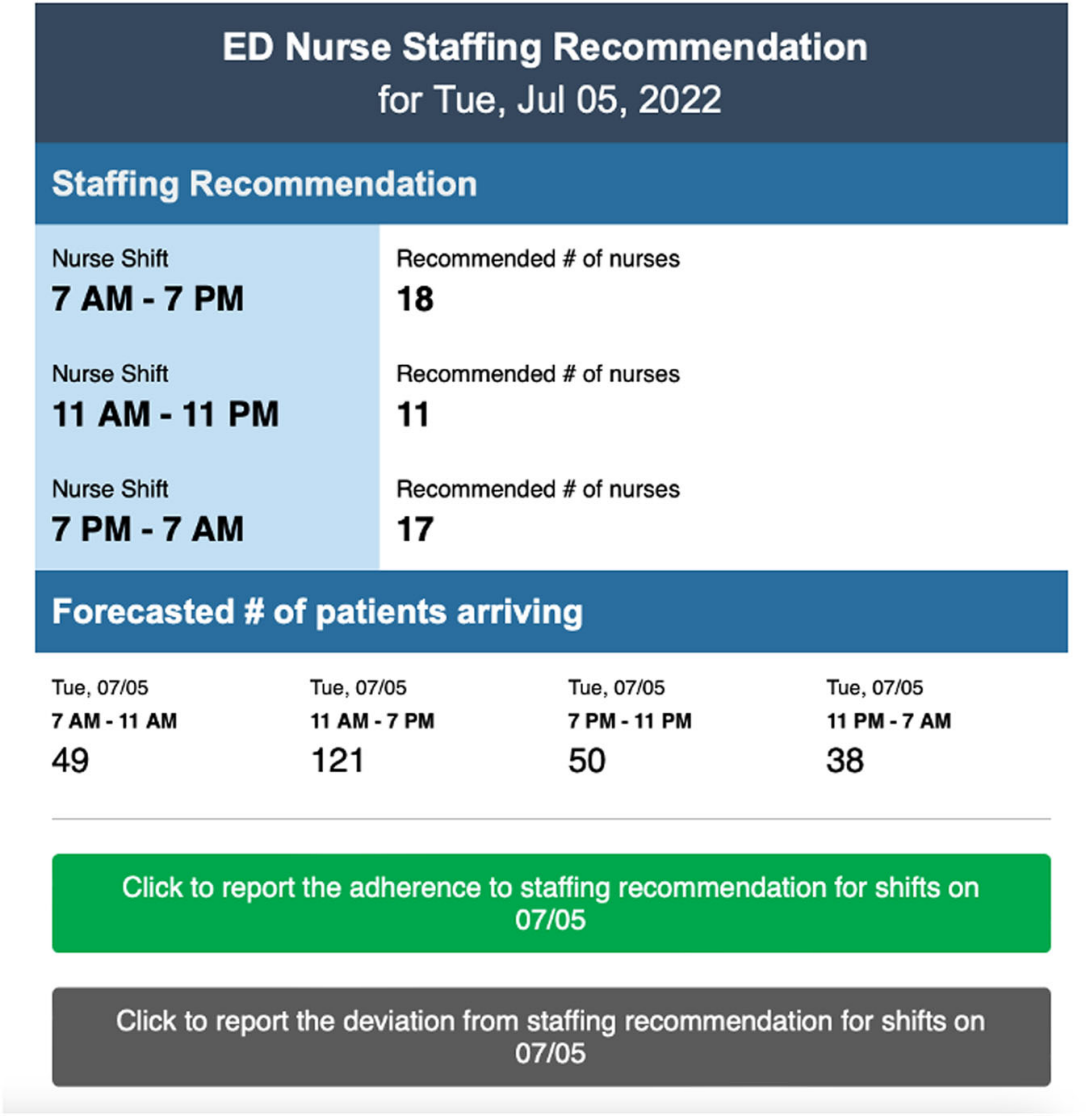
Example email during the experiment stage sent on July 4, 2022.

**Fig. 7:**
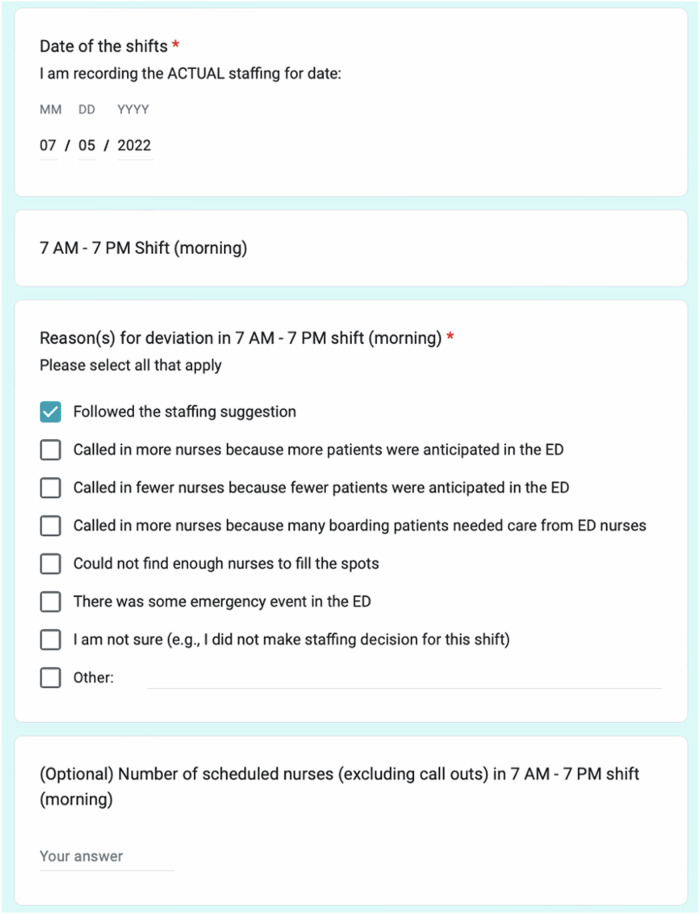
Example adherence/deviation survey linked to the email sent on July 4, 2022.

**Fig. 8:**
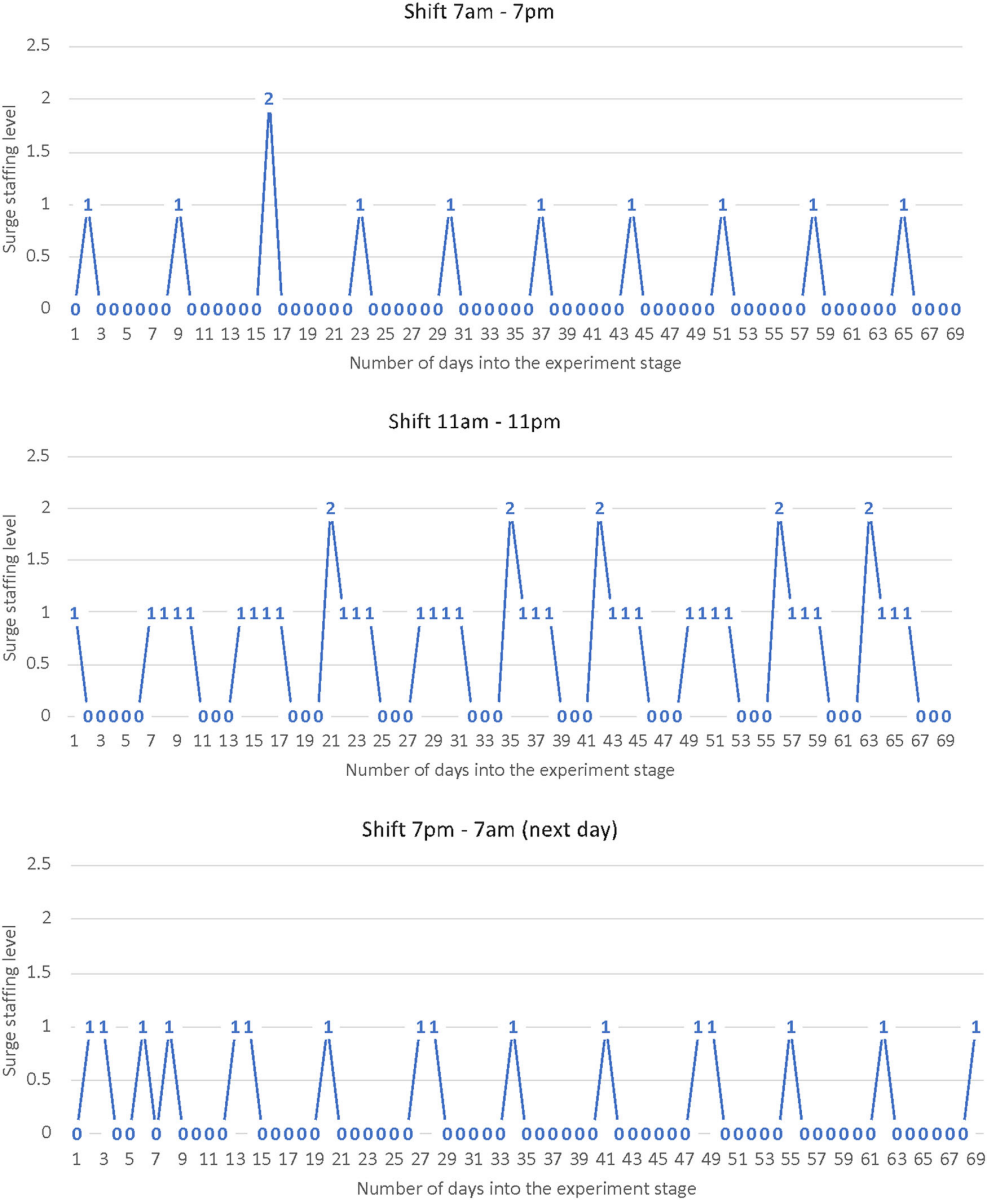
Recommended surge staffing levels during the experiment stage.

Following the control stage, the experiment stage lasted 69 days, from June 28, 2022, to September 4, 2022. This phase marked the actual implementation of the prediction-driven nurse staffing system. Figure 5 illustrates the daily routines for the implementation. Each day:

- 7 AM **–** 10 AM: the hospital informatics team recorded recent patient arrival counts into the system for use as inputs for the surge-stage prediction model.
- 10 AM **–** 11 AM: our system, developed using Google Apps Script, generated forecasts of patient arrival counts and staffing recommendations based on the predictions for the following day (starting from 7 am).
- 11 AM **–** 12 PM: the ED nursing leadership received an email (Fig. 6) containing the forecasted patient arrival counts, staffing recommendations, and links for reporting adherence to or deviation from the staffing recommendations (Fig. 7). Upon receiving the email, the nursing leadership would recruit additional nurses (Fig. 8) with incentive pay if the recommendation from our system surpassed the planned staffing level for the next day. The staffing algorithm guaranteed that the recommended staffing level was always at or above the planned staffing level. Nursing leadership then utilized the links in the email to report whether the staffing recommendations were followed and, if not, the reasons for not following the recommendations.

The step-wise construction and application of the prediction-driven staffing framework for our hospital’s pilot study are provided in Supplementary Note 1. The following sections focus on evaluating the efficacy of the pilot.

### Data and metrics for pilot evaluation

To assess the efficacy of the prediction-driven staffing framework, we compared patient throughput metrics and staffing costs across the control and experiment stages from May 2, 2022, to September 4, 2022. We utilized data from the electronic health records and staff payroll system. We applied a set of techniques to compare the system performance between the control and experiment phases, including summary statistics and regression analyses. In addition, we compared time-series data related to workload and staffing, and constructed quality-versus-efficiency tradeoff curves. Details on each metric are provided below.

### Summary statistics across the control and experimental stages

Four categories of summary statistics regarding patient characteristics and system operations were analyzed. First, summary statistics were computed for patient volume, age, gender, arrival means, acuity levels, and insurance types. Second, the average number of nurses per hour and surge nurses called in were calculated. Third, performance metrics for throughput were calculated, including average door-to-evaluation, active treatment (defined as the duration between medical screening exam and disposition decision), boarding, LOS in the ED, and LWBS rate. Fourth, to contextualize staffing composition across the pilot period, we examined the experience levels of nurses in the ED at the beginning of the control and experiment stages. Two-sample Kolmogorov-Smirnov (KS) tests were conducted to examine whether these statistics significantly differed between the control and experiment stages.

### Time-series comparison of ED census and staffing levels

The degree of alignment between staffing levels and concurrent patient care needs in the ED was assessed. In particular, we compared the hourly ED census and staffing levels (multiplied by a nurse-to-patient ratio of 1-to-4) in the control and experiment stages. The census was defined as

1 $${\rm{C}}{\rm{e}}{\rm{n}}{\rm{s}}{\rm{u}}{\rm{s}}=\,{\rm{n}}{\rm{u}}{\rm{m}}{\rm{b}}{\rm{e}}{\rm{r}}\,{\rm{o}}{\rm{f}}\,{\rm{w}}{\rm{a}}{\rm{i}}{\rm{t}}{\rm{i}}{\rm{n}}{\rm{g}}\,{\rm{p}}{\rm{a}}{\rm{t}}{\rm{i}}{\rm{e}}{\rm{n}}{\rm{t}}{\rm{s}}+{\rm{n}}{\rm{u}}{\rm{m}}{\rm{b}}{\rm{e}}{\rm{r}}\,{\rm{o}}{\rm{f}}\,{\rm{p}}{\rm{a}}{\rm{t}}{\rm{i}}{\rm{e}}{\rm{n}}{\rm{t}}{\rm{s}}\,{\rm{i}}{\rm{n}}\,{\rm{t}}{\rm{r}}{\rm{e}}{\rm{a}}{\rm{t}}{\rm{m}}{\rm{e}}{\rm{n}}{\rm{t}}+{\rm{n}}{\rm{u}}{\rm{m}}{\rm{b}}{\rm{e}}{\rm{r}}\,{\rm{o}}{\rm{f}}\,{\rm{b}}{\rm{o}}{\rm{a}}{\rm{r}}{\rm{d}}{\rm{i}}{\rm{n}}{\rm{g}}\,{\rm{p}}{\rm{a}}{\rm{t}}{\rm{i}}{\rm{e}}{\rm{n}}{\rm{t}}{\rm{s}}/3$$

In the census calculation in Eq. (1), we divided the number of boarding patients by 3 to reflect the assumption that boarding patients required, on average, one-third of nursing care compared to patients awaiting disposition decisions. The number of waiting patients was included to account for pending care needs if there were sufficient capacity. To quantify the overall disparity between census and staffing levels, we calculated the mean squared difference between these two measures. A smaller mean squared difference value indicated a higher efficacy in aligning nurse staffing with patients’ care needs.

### Tradeoff curves

We examined how the traditional staffing and the new prediction-driven staffing balanced access-to-care targets and staffing costs by constructing “tradeoff curves.” Specifically, for each day during the control and experiment stages, we computed the total staffing cost, average patient waiting time, and LWBS rate. We then plotted the staffing cost against the average waiting time or LWBS rate to quantify the tradeoffs between cost and access to care during the control and experiment stage. For a fixed staffing cost, the staffing practice associated with a lower tradeoff curve was more likely to achieve a lower average patient waiting time or a smaller LWBS rate. Conversely, when targeting a specific access-to-care goal, the staffing rule with a lower tradeoff curve could attain it with a lower staffing cost.

### Differences in staffing efficiency across the control and experimental stages

We also studied the difference in staffing efficiency through regression analysis. This allowed us to control for other related covariates. Specifically, we divided each day into two consecutive 12-h time windows: 7 am – 7 pm and 7 pm – 7 am (next day). Each period in each day was an observation in our regression analysis. The dependent/outcome variable was the hourly staffing cost for the 12-hour window. We controlled for several variables that could affect the staffing cost. The first set of control variables accounted for temporal variations, e.g., day-of-the-week and time-of-the-day effects. The second set of control variables captured *existing* care needs (from the previous time window), measured by the number of patients waiting, in treatment, and boarding at the beginning of the period (e.g., 7 am or 7 pm). The third set of control variables captured care needs from *new* patient arrivals during the period, including the average hourly patient arrival count and the average LOS for patients arriving within the time window. The fourth set of covariates quantified the access-to-care metrics, including the average patient waiting time and the LWBS rate during the period. Lastly, we added a binary indicator indicating whether the time window was in the experiment stage. Notably, the fitted coefficient associated with the experiment-stage indicator quantified the difference in hourly staffing costs between the control and experiment stages while keeping all other factors (e.g., patient care needs and access-to-care measures) constant. To ensure the robustness of the results, we also conducted sensitivity analyses for different time window lengths: 4 h, 2 h, and 1 h (instead of 12 h).

### Impact of not following the staffing levels recommended by the system

We computed the difference between the actual and recommended staffing levels during the experiment stage to understand the impact of deviations from recommended staffing levels. To this end, we segmented each day into two consecutive 12-hour time intervals starting from 7 am and 7 pm and calculated the staffing deviation for each interval in Eq. (2) as

2 $${\rm{D}}{\rm{e}}{\rm{v}}{\rm{i}}{\rm{a}}{\rm{t}}{\rm{i}}{\rm{o}}{\rm{n}}=({\rm{a}}{\rm{c}}{\rm{t}}{\rm{u}}{\rm{a}}{\rm{l}}\,{\rm{t}}{\rm{o}}{\rm{t}}{\rm{a}}{\rm{l}}\,{\rm{n}}{\rm{u}}{\rm{r}}{\rm{s}}{\rm{e}}\,{\rm{h}}{\rm{o}}{\rm{u}}{\rm{r}}{\rm{s}}-{\rm{r}}{\rm{e}}{\rm{c}}{\rm{o}}{\rm{m}}{\rm{m}}{\rm{e}}{\rm{n}}{\rm{d}}{\rm{e}}{\rm{d}}\,{\rm{t}}{\rm{o}}{\rm{t}}{\rm{a}}{\rm{l}}\,{\rm{n}}{\rm{u}}{\rm{r}}{\rm{s}}{\rm{e}}\,{\rm{h}}{\rm{o}}{\rm{u}}{\rm{r}}{\rm{s}})/12,$$

which could be interpreted as the nurse count deviation per hour. We first calculated summary statistics for these deviation values to understand the overall pattern. We then conducted a patient-level regression analysis to quantify the impact of the deviations on patients’ waiting times. Specifically, for each patient entering the experimental stage, we regressed their waiting time against the nurse staffing deviation during the time window at which the patient arrived and two indicators indicating whether the actual staffing level for the time window exceeded or fell below the recommendation by more than 20%. We also controlled for additional covariates, including temporal variations, patient-level characteristics, concurrent system congestion status, and staffing levels.

## Supplementary information

Supplementary Information

## Data Availability

The non-identifiable data that support the findings of this study are available on request from the corresponding author. The data are not publicly available due to privacy or ethical restrictions.The code supporting this study is available upon request from the corresponding author.

## Code availability

The code supporting this study is available upon request from the corresponding author.
