# Supplementary material for: Implementing a prediction driven framework for emergency department nurse staffing to optimize real time decisions
Source: Npj Health Syst. 2025 May 8;2:16. doi: 10.1038/s44401-025-00019-2 (PMC13354205; doi:10.1038/s44401-025-00019-2)
Supplement: Supplementary file 1 — Supplementary Information [file 44401_2025_19_MOESM1_ESM.docx]

**Supplementary Information for “Implementing a Prediction Driven Framework for Emergency Department Nurse Staffing to Optimize Real Time Decisions”**

**Supplementary Note 1. Prediction-Driven Staffing Framework**

The prediction-driven staffing framework consisted of two fundamental components: 1) a two-stage prediction model that forecasted upcoming ED arrivals, and 2) a two-stage staffing algorithm that mapped the predicted arrivals to appropriate staffing levels. The subsequent sections provide a thorough overview of the construction of each component.

**Data for developing prediction-driven staffing framework**

To construct the prediction-driven staffing framework, we leveraged data from 1) patient electronic health records, and 2) weather data[1] published by the National Centers for Environmental Information. The entire data spanned from February 1, 2019, to May 17, 2022. However, we deliberately omitted the data records between February 1, 2020, and March 1, 2022, to mitigate the impact of the COVID-19 pandemic, thereby ensuring more accurate representation of staffing needs unaffected by the exceptional circumstances arising from the pandemic. Throughout the subsequent sections of the paper, we will denote the one-year data set from February 1, 2019, to January 31, 2020, as the pre-COVID data set. Additionally, the more recent dataset, spanning from March 2, 2022, to May 17, 2022, will be referred to as the post-COVID data set. The data were processed into shift-level features regarding patient arrivals, day of the week, time of shift, near-holiday indicators, and weather conditions. These shift-level features were then used for two purposes: firstly, to develop a two-stage prediction model for ED arrivals, following similar procedures in Hu et al.[2]; and secondly, to calibrate relevant parameters in the two-stage staffing algorithm proposed by Hu et al.[3].

**Two-stage prediction model**

The two-stage prediction model aimed to forecast ED arrivals in two distinct time epochs, synchronized with the specific moments when base and surge staffing decisions were finalized. In our hospital, the base staffing decisions for a nursing shift were typically made four weeks in advance, and the surge staffing decisions were made approximately one day prior to the beginning of the nursing shift.

When constructing the base-stage prediction model, due to the required lead time of four weeks, we worked with a relatively limited number of predictors to ensure the model's feasibility within the specified time frame. Specifically, we developed a linear regression model that incorporated categorical variables to differentiate the day of the week and the time of the shift. Because of its relatively simple structure, we trained the model on the post-COVID data set (which could be considered as the training data for the base-stage prediction model) to capture more recent temporal variations of ED demand. The fitted coefficients of the base-stage linear regression model are listed in Supplementary Table 1. Furthermore, as shown in Supplementary Table 3, in terms of prediction accuracy, the model achieved a root mean square error (RMSE) of 8.161 and mean absolute percentage error (MAPE) of 13.066% on the training data set. During the subsequent experiment stage, the model achieved an RMSE of 11.261 and MAPE of 13.414%, which could be considered as its prediction accuracy on a proper “test” data set.

In contrast to the base-stage prediction model, the surge-stage prediction model was able to leverage more real-time information as it was executed at a time epoch much closer to the beginning of the nursing shift. As a result, we constructed the surge-stage prediction model using linear regression with additional real-time covariates. Following procedures similar to those outlined in Hu et al.[2], we first identified and selected statistically significant covariates. For this model selection process, we used the pre-COVID data set as training data and the post-COVID data set as test data. Once the covariates were finalized, the selected model was then trained on the entire dataset, combining both the pre-COVID and post-COVID data sets, to obtain the fitted coefficients. The covariates incorporated into the finalized surge-stage prediction model included the day of the week, time of the day, near-holiday indicators, weather conditions, and the 21-day moving average of arrival counts during shifts occurring at the same time of day (e.g., 7am – 11am, 11am – 7pm, 7pm – 11pm, or 11pm – 7am the next day). The fitted coefficients of the surge-stage linear regression model are listed in Supplementary Table 2. Moreover, in the terms of prediction accuracy, the model achieved an RMSE of 8.558 and MAPE of 13.003% on the training data set (which combined the pre-COVID and post-COVID data sets). Furthermore, during the experiment stage, the model achieved an RMSE of 9.973 and MAPE of 12.126%.

Supplementary Table 3 provides a comparison of prediction accuracy between the base-stage and surge-stage prediction models, evaluated on both the training and posterior experiment-stage (test) data. Notably, the surge-stage prediction model demonstrated substantial improvement in prediction accuracy on the posterior data from the experiment stage. Specifically, after incorporating the additional real-time covariates, the surge-stage prediction model achieved a 11.438% reduction in RMSE and a 9.062% decrease in MAPE.

Finally, we highlighted that the surge-stage prediction model incorporated a critical predictor: the 21-day moving average of arrival counts during shifts occurring at the same time of day, which required input of the most recent patient arrival counts. As illustrated in the daily routine diagram in Figure 1 of the main paper, during the experiment stage, the surge-stage prediction model was executed on a daily rolling basis between 10am and 11am to forecast patient arrivals for the next day. To facilitate the timely population of the moving-average predictor, our informatics team reported the arrival counts from the day before (and up to 7am of today) sometime between 7am and 10am. This meticulous daily input process, while essential, was susceptible to understandable human errors, such as missing data reporting. To bolster the robustness of surge-stage execution, we trained 6 alternative surge-stage prediction models. These alternative models were designed to accommodate missing daily inputs for up to 6 days, as elaborated in the last section of this note. This deliberate design feature enhanced the reliability and resilience of our system.

**Supplementary Table 1. Base-Stage Linear Regression Model for Base Staffing**

| Covariate | Coefficient | Standard error | P-value |
| --- | --- | --- | --- |
| Intercept | 54.909 | 2.581 | < 0.001 |
| Monday 11am – 7pm | 72.818 | 3.649 | < 0.001 |
| Monday 7pm – 11pm | -7.636 | 3.649 | 0.037 |
| Monday 11pm – 7am (next day) | -22.818 | 3.649 | < 0.001 |
| Tuesday 7am – 11am | -12.364 | 3.649 | < 0.001 |
| Tuesday 11am – 7pm | 66.273 | 3.649 | < 0.001 |
| Tuesday 7pm – 11pm | -10.545 | 3.649 | 0.004 |
| Tuesday 11pm – 7am (next day) | -21.818 | 3.649 | < 0.001 |
| Wednesday 7am – 11am | -12.182 | 3.649 | < 0.001 |
| Wednesday 11am – 7pm | 60.727 | 3.649 | < 0.001 |
| Wednesday 7pm – 11pm | -12.000 | 3.649 | 0.001 |
| Wednesday 11pm – 7am (next day) | -21.455 | 3.649 | < 0.001 |
| Thursday 7am – 11am | -11.909 | 3.649 | 0.001 |
| Thursday 11am – 7pm | 54.545 | 3.649 | < 0.001 |
| Thursday 7pm – 11pm | -9.273 | 3.649 | 0.012 |
| Thursday 11pm – 7am (next day) | -21.636 | 3.649 | < 0.001 |
| Friday 7am – 11am | -12.091 | 3.649 | 0.001 |
| Friday 11am – 7pm | 54.818 | 3.649 | < 0.001 |
| Friday 7pm – 11pm | -10.000 | 3.649 | 0.007 |
| Friday 11pm – 7am (next day) | -17.091 | 3.649 | < 0.001 |
| Saturday 7am – 11am | -20.091 | 3.649 | < 0.001 |
| Saturday 11am – 7pm | 44.455 | 3.649 | < 0.001 |
| Saturday 7pm – 11pm | -14.909 | 3.649 | < 0.001 |
| Saturday 11pm – 7am (next day) | -15.364 | 3.649 | < 0.001 |
| Sunday 7am – 11am | -18.455 | 3.649 | < 0.001 |
| Sunday 11am – 7pm | 42.727 | 3.649 | < 0.001 |
| Sunday 7pm – 11pm | -13.545 | 3.649 | < 0.001 |
| Sunday 11pm – 7am (next day) | -18.909 | 3.649 | < 0.001 |

**Supplementary Table 2. Surge-Stage Linear Regression Model for Surge Staffing**

| Covariate | Coefficient | Standard error | P-value |
| --- | --- | --- | --- |
| Intercept | 34.894 | 3.732 | < 0.001 |
| Monday 11am – 7pm | 45.982 | 6.149 | < 0.001 |
| Monday 7pm – 11pm | -5.378 | 1.560 | < 0.001 |
| Monday 11pm – 7am (next day) | -15.071 | 1.630 | < 0.001 |
| Tuesday 7am – 11am | -9.285 | 1.552 | < 0.001 |
| Tuesday 11am – 7pm | 33.123 | 6.163 | < 0.001 |
| Tuesday 7pm – 11pm | -8.581 | 1.574 | < 0.001 |
| Tuesday 11pm – 7am (next day) | -17.293 | 1.638 | < 0.001 |
| Wednesday 7am – 11am | -9.318 | 1.542 | < 0.001 |
| Wednesday 11am – 7pm | 32.260 | 6.142 | < 0.001 |
| Wednesday 7pm – 11pm | -8.005 | 1.562 | < 0.001 |
| Wednesday 11pm – 7am (next day) | -15.877 | 1.635 | < 0.001 |
| Thursday 7am – 11am | -9.786 | 1.543 | < 0.001 |
| Thursday 11am – 7pm | 27.523 | 6.150 | < 0.001 |
| Thursday 7pm – 11pm | -8.542 | 1.563 | < 0.001 |
| Thursday 11pm – 7am (next day) | -15.416 | 1.633 | < 0.001 |
| Friday 7am – 11am | -12.648 | 1.540 | < 0.001 |
| Friday 11am – 7pm | 28.312 | 6.148 | < 0.001 |
| Friday 7pm – 11pm | -8.532 | 1.560 | < 0.001 |
| Friday 11pm – 7am (next day) | -12.469 | 1.630 | < 0.001 |
| Saturday 7am – 11am | -17.924 | 1.545 | < 0.001 |
| Saturday 11am – 7pm | 16.578 | 6.145 | 0.007 |
| Saturday 7pm – 11pm | -11.681 | 1.566 | < 0.001 |
| Saturday 11pm – 7am (next day) | -9.887 | 1.635 | < 0.001 |
| Sunday 7am – 11am | -16.433 | 1.545 | < 0.001 |
| Sunday 11am – 7pm | 19.048 | 6.152 | 0.002 |
| Sunday 7pm – 11pm | -12.529 | 1.566 | < 0.001 |
| Sunday 11pm – 7am (next day) | -13.529 | 1.635 | < 0.001 |
| Holiday | -8.697 | 1.419 | < 0.001 |
| Holiday +1 day | 3.936 | 1.423 | 0.006 |
| 21-day moving average of arrivals during same type of shift | 0.434 | 0.085 | < 0.001 |
| Is raining | -0.514 | 0.426 | 0.228 |
| Max temperature exceeds 30$℃$ | -0.398 | 0.610 | 0.514 |

**Supplementary Table 3. Prediction Accuracy of the Prediction Models**

|  | Training RMSE | Training MAPE | Experiment RMSE | Experiment MAPE |
| --- | --- | --- | --- | --- |
| Base-stage prediction model | 8.161 | 13.066% | 11.261 | 13.414% |
| Surge-stage prediction model | 8.558 | 13.003% | 9.973 | 12.126% |

**Alternative surge-stage prediction models**

To complement the primary linear regression model used in our surge-stage prediction framework, we evaluated two additional ML models, XGBoost and ANN, to assess their predictive performance. These models were developed solely to examine their prediction performance and were not used in the actual pilot for staffing. This section details the development procedures, hyperparameter tuning methodologies, and final selected hyperparameters for these alternative models.

The XGBoost and ANN models were trained using the same dataset as the linear regression model, as described above. These models were then tested on the arrival data during the experiment stage of the pilot to evaluate their predictive performance. To ensure a fair comparison, we employed nested cross-validation, with an inner loop for hyperparameter tuning using Bayesian optimization and an outer loop for model evaluation using k-fold cross-validation (k = 5). Model performance was assessed using RMSE and MAPE.

For the XGBoost model, we conducted an extensive hyperparameter search using grid search over multiple configurations. The grid search explored combinations of learning rates, tree depths, the number of boosting rounds, and regularization terms. The search space included learning rates between 0.1 and 0.5, maximum depths ranging from 1 to 8, and boosting rounds spanning from 10 to 200 in increments of 10. Additionally, L1 (reg_alpha) and L2 (reg_lambda) regularization terms were tuned across values from 0.2 to 1.0. The best-performing model was selected based on validation RMSE, and the final chosen hyperparameters were a learning rate of 0.1, a max depth of 3, 180 estimators, an L1 regularization term (reg_alpha) of 0.6, and an L2 regularization term (reg_lambda) of 0.8. The model was trained using all available CPU cores (n_jobs = -1) for efficient parallel processing.

For the ANN model, we used Keras with TensorFlow backend and applied hyperparameter tuning using Keras Tuner. The model architecture was dynamically selected based on the tuning process. The input layer consisted of features from X_train.shape[1], followed by a variable number of hidden layers (between 2 and 4). Each hidden layer had between 32 and 128 neurons, with either ReLU or LeakyReLU activation, as determined by the tuning process. Batch normalization was applied after each hidden layer, and dropout rates ranged from 0.1 to 0.3, also selected through tuning. The output layer consisted of a single neuron with a linear activation function to predict continuous values. The optimizer was chosen between Adam and RMSprop based on tuning, and the model was compiled with mean squared error (MSE) as the loss function and mean absolute error (MAE) as the evaluation metric. Hyperparameter tuning was conducted over 50 trials using a batch size of 64 and 50 epochs per trial. After selecting the best configuration, the final ANN model was trained for 100 epochs with a batch size of 64.

After training and validation, we compared the prediction accuracy of all models using test data from the experiment stage. Recall that the surge-stage linear regression achieved an RMSE of 8.558 in training and 9.973 in testing, with a MAPE of 13.003% in training and 12.126% in testing. The XGBoost model performed better in training with an RMSE of 6.071 and a MAPE of 10.147%, but in testing, it had an RMSE of 10.800 and a MAPE of 12.981%. The ANN model had an RMSE of 8.304 in training and 10.555 in testing, with a MAPE of 13.176% in training and 12.791% in testing.

The results indicated that while XGBoost provided a lower RMSE in training, it did not generalize as well in testing, resulting in a higher RMSE compared to the linear regression model. Similarly, the ANN model did not yield meaningful improvements, likely due to overfitting on training data despite dropout regularization. Given the marginal gains and interpretability benefits, the linear regression model remained the primary choice for deployment for our use case. However, future research could explore hybrid models or alternative feature engineering techniques to further enhance predictive accuracy.

**Two-stage staffing algorithm**

After developing the two-stage prediction model, we proceeded to implement a two-stage staffing algorithm developed by Hu et al.[3]. This algorithm effectively translated the predicted patient arrivals into appropriate staffing levels. In our hospital, each nursing shift entailed two staffing decisions in different time scales. The base staffing levels were finalized four weeks in advance, based on relatively crude demand forecast generated by the base-stage prediction model. The surge staff could be called in approximately one day before the shift, guided by more refined demand forecast provided by the surge-stage prediction model.

***Base staffing***

In determining the base staffing levels, the base-stage prediction model only differentiated the day-of-the-week and time-of-the-day effects. Consequently, the base staffing levels similarly accounted for temporal variations within each week and maintained identical across different weeks. The specific values for the implemented base staffing levels are listed in Table 1 of the main paper. These numerical values were derived using procedures similar to those outlined in Hu et al.[3]. Intuitively, the base staffing levels should address the majority of patient needs calibrated from historical averages from the data. In addition, the staffing levels were further adjusted to 1) accommodate patient handoffs at shift transitions (see Section 7.4.2 in Hu et al.[3]), and 2) include non-patient-facing roles, such as charge, triage, ambulance triage, trauma, and floating nurses, in accordance with the clinical guidelines of our hospital. To be timely implemented during the experiment stage from June 28, 2022, to September 4, 2022, this base staffing plan was confirmed on May 16, 2022.

***Surge staffing***

The integration of additional real-time predictors into the surge-stage prediction model resulted in variable surge staffing levels across shifts, which depended on the concurrent local conditions and weather dynamics. During each day in the experiment stage, we employed the surge-stage prediction model to forecast the number of patient arrivals for the following day. These predictions were overall more accurate than the demand forecasts generated by the base-stage prediction model. After obtaining the updated demand forecasts from the surge-stage prediction model, we applied the staffing algorithm developed by Hu et al.[3] to compute the updated staffing levels for the upcoming nursing shifts. Subsequently, the updated demand forecasts and staffing levels were communicated to ED nursing leadership through an email generated by the Google Apps Script. Recall that Figure 2 in the main paper exemplified such an example sent on July 4, 2022, in preparation for the upcoming nursing shifts on July 5, 2022. Upon receipt of the email, the nursing leadership compared the updated staffing recommendation with the existing base staffing level. When the updated staffing recommendation exceeded the base staffing levels, the nursing leadership proactively tried to meet the new staffing suggestions outlined in the email by recruiting additional nurses and offering incentive pays. For example, in Figure 2 of the main paper, the updated staffing recommendation was 18 nurses for the 7am – 7pm shift, 11 nurses for the 11am – 11pm shift, and 17 nurses for the 7pm – 7am shift. In comparison, the base staffing levels listed in Table 1 of the main paper had 18, 10, and 16 base nurses staffed for these respective shifts. To bridge the gap between the existing base staffing levels and the updated suggestions, the nursing leadership aimed to call in 1 additional surge nurse for each of the latter two shifts during the day. Throughout the experiment stage, the recommended surge staffing levels (Figure 4 in the main paper) stayed relatively low, with an average of 0.159, 0.609, and 0.232 additional nurses for the three nursing shifts per day.

**Alternative surge-stage prediction models to accommodate missing daily data inputs**

**Supplementary Table 4. Surge-Stage Prediction Model (Missing 1 Day of Input)**

| Covariate | Coefficient | Standard error | P-value |
| --- | --- | --- | --- |
| Intercept | 35.936 | 3.739 | < 0.001 |
| Monday 11am – 7pm | 47.671 | 6.171 | < 0.001 |
| Monday 7pm – 11pm | -5.309 | 1.562 | < 0.001 |
| Monday 11pm – 7am (next day) | -15.233 | 1.631 | < 0.001 |
| Tuesday 7am – 11am | -9.269 | 1.553 | < 0.001 |
| Tuesday 11am – 7pm | 34.823 | 6.183 | < 0.001 |
| Tuesday 7pm – 11pm | -8.511 | 1.576 | < 0.001 |
| Tuesday 11pm – 7am (next day) | -17.445 | 1.640 | < 0.001 |
| Wednesday 7am – 11am | -9.341 | 1.543 | < 0.001 |
| Wednesday 11am – 7pm | 33.940 | 6.165 | < 0.001 |
| Wednesday 7pm – 11pm | -7.951 | 1.564 | < 0.001 |
| Wednesday 11pm – 7am (next day) | -16.049 | 1.636 | < 0.001 |
| Thursday 7am – 11am | -9.798 | 1.544 | < 0.001 |
| Thursday 11am – 7pm | 29.223 | 6.170 | < 0.001 |
| Thursday 7pm – 11pm | -8.476 | 1.565 | < 0.001 |
| Thursday 11pm – 7am (next day) | -15.574 | 1.635 | < 0.001 |
| Friday 7am – 11am | -12.655 | 1.541 | < 0.001 |
| Friday 11am – 7pm | 29.987 | 6.173 | < 0.001 |
| Friday 7pm – 11pm | -8.475 | 1.562 | < 0.001 |
| Friday 11pm – 7am (next day) | -12.634 | 1.631 | < 0.001 |
| Saturday 7am – 11am | -17.951 | 1.547 | < 0.001 |
| Saturday 11am – 7pm | 18.273 | 6.166 | 0.003 |
| Saturday 7pm – 11pm | -11.626 | 1.568 | < 0.001 |
| Saturday 11pm – 7am (next day) | -10.067 | 1.635 | < 0.001 |
| Sunday 7am – 11am | -16.459 | 1.547 | < 0.001 |
| Sunday 11am – 7pm | 20.755 | 6.171 | < 0.001 |
| Sunday 7pm – 11pm | -12.477 | 1.569 | < 0.001 |
| Sunday 11pm – 7am (next day) | -13.701 | 1.635 | < 0.001 |
| Holiday | -8.776 | 1.421 | < 0.001 |
| Holiday +1 day | 3.752 | 1.425 | 0.009 |
| 21-day moving average of arrivals during same-type shifts (lag 2) | 0.410 | 0.086 | < 0.001 |
| Is raining | -0.506 | 0.427 | 0.236 |
| Max temperature exceeds 30$℃$ | -0.412 | 0.611 | 0.501 |

**Supplementary Table 5. Surge-Stage Prediction Model (Missing 2 Days of Input)**

| Covariate | Coefficient | Standard error | P-value |
| --- | --- | --- | --- |
| Intercept | 36.235 | 3.744 | < 0.001 |
| Monday 11am – 7pm | 48.209 | 6.172 | < 0.001 |
| Monday 7pm – 11pm | -5.282 | 1.562 | < 0.001 |
| Monday 11pm – 7am (next day) | -15.272 | 1.632 | < 0.001 |
| Tuesday 7am – 11am | -9.244 | 1.553 | < 0.001 |
| Tuesday 11am – 7pm | 35.344 | 6.188 | < 0.001 |
| Tuesday 7pm – 11pm | -8.467 | 1.575 | < 0.001 |
| Tuesday 11pm – 7am (next day) | -17.474 | 1.641 | < 0.001 |
| Wednesday 7am – 11am | -9.324 | 1.544 | < 0.001 |
| Wednesday 11am – 7pm | 34.455 | 6.171 | < 0.001 |
| Wednesday 7pm – 11pm | -7.923 | 1.564 | < 0.001 |
| Wednesday 11pm – 7am (next day) | -16.089 | 1.637 | < 0.001 |
| Thursday 7am – 11am | -9.795 | 1.544 | < 0.001 |
| Thursday 11am – 7pm | 29.736 | 6.176 | < 0.001 |
| Thursday 7pm – 11pm | -8.447 | 1.565 | < 0.001 |
| Thursday 11pm – 7am (next day) | -15.612 | 1.636 | < 0.001 |
| Friday 7am – 11am | -12.650 | 1.542 | < 0.001 |
| Friday 11am – 7pm | 30.507 | 6.178 | < 0.001 |
| Friday 7pm – 11pm | -8.441 | 1.562 | < 0.001 |
| Friday 11pm – 7am (next day) | -12.663 | 1.633 | < 0.001 |
| Saturday 7am – 11am | -17.939 | 1.547 | < 0.001 |
| Saturday 11am – 7pm | 18.772 | 6.175 | 0.002 |
| Saturday 7pm – 11pm | -11.599 | 1.569 | < 0.001 |
| Saturday 11pm – 7am (next day) | -10.106 | 1.636 | < 0.001 |
| Sunday 7am – 11am | -16.459 | 1.547 | < 0.001 |
| Sunday 11am – 7pm | 21.283 | 6.174 | < 0.001 |
| Sunday 7pm – 11pm | -12.446 | 1.569 | < 0.001 |
| Sunday 11pm – 7am (next day) | -13.748 | 1.636 | < 0.001 |
| Holiday | -8.762 | 1.421 | < 0.001 |
| Holiday +1 day | 3.671 | 1.425 | 0.010 |
| 21-day moving average of arrivals during same-type shifts (lag 3) | 0.403 | 0.086 | < 0.001 |
| Is raining | -0.498 | 0.427 | 0.243 |
| Max temperature exceeds 30$℃$ | -0.421 | 0.611 | 0.491 |

**Supplementary Table 6. Surge-Stage Prediction Model (Missing 3 Days of Input)**

| Covariate | Coefficient | Standard error | P-value |
| --- | --- | --- | --- |
| Intercept | 35.824 | 3.744 | < 0.001 |
| Monday 11am – 7pm | 47.543 | 6.167 | < 0.001 |
| Monday 7pm – 11pm | -5.302 | 1.561 | < 0.001 |
| Monday 11pm – 7am (next day) | -15.211 | 1.632 | < 0.001 |
| Tuesday 7am – 11am | -9.249 | 1.553 | < 0.001 |
| Tuesday 11am – 7pm | 34.680 | 6.182 | < 0.001 |
| Tuesday 7pm – 11pm | -8.498 | 1.575 | < 0.001 |
| Tuesday 11pm – 7am (next day) | -17.411 | 1.641 | < 0.001 |
| Wednesday 7am – 11am | -9.313 | 1.543 | < 0.001 |
| Wednesday 11am – 7pm | 33.774 | 6.169 | < 0.001 |
| Wednesday 7pm – 11pm | -7.936 | 1.563 | < 0.001 |
| Wednesday 11pm – 7am (next day) | -16.023 | 1.636 | < 0.001 |
| Thursday 7am – 11am | -9.781 | 1.544 | < 0.001 |
| Thursday 11am – 7pm | 29.051 | 6.175 | < 0.001 |
| Thursday 7pm – 11pm | -8.473 | 1.565 | < 0.001 |
| Thursday 11pm – 7am (next day) | -15.548 | 1.635 | < 0.001 |
| Friday 7am – 11am | -12.649 | 1.541 | < 0.001 |
| Friday 11am – 7pm | 29.811 | 6.179 | < 0.001 |
| Friday 7pm – 11pm | -8.470 | 1.562 | < 0.001 |
| Friday 11pm – 7am (next day) | -12.603 | 1.632 | < 0.001 |
| Saturday 7am – 11am | -17.938 | 1.547 | < 0.001 |
| Saturday 11am – 7pm | 18.091 | 6.173 | 0.003 |
| Saturday 7pm – 11pm | -11.624 | 1.568 | < 0.001 |
| Saturday 11pm – 7am (next day) | -10.037 | 1.636 | < 0.001 |
| Sunday 7am – 11am | -16.452 | 1.547 | < 0.001 |
| Sunday 11am – 7pm | 20.584 | 6.176 | < 0.001 |
| Sunday 7pm – 11pm | -12.475 | 1.568 | < 0.001 |
| Sunday 11pm – 7am (next day) | -13.684 | 1.636 | < 0.001 |
| Holiday | -8.808 | 1.421 | < 0.001 |
| Holiday +1 day | 3.677 | 1.425 | 0.010 |
| 21-day moving average of arrivals during same-type shifts (lag 4) | 0.413 | 0.086 | < 0.001 |
| Is raining | -0.488 | 0.427 | 0.254 |
| Max temperature exceeds 30$℃$ | -0.435 | 0.611 | 0.477 |

**Supplementary Table 7. Surge-Stage Prediction Model (Missing 4 Days of Input)**

| Covariate | Coefficient | Standard error | P-value |
| --- | --- | --- | --- |
| Intercept | 36.818 | 3.739 | < 0.001 |
| Monday 11am – 7pm | 49.158 | 6.168 | < 0.001 |
| Monday 7pm – 11pm | -5.238 | 1.563 | < 0.001 |
| Monday 11pm – 7am (next day) | -15.362 | 1.632 | < 0.001 |
| Tuesday 7am – 11am | -9.257 | 1.554 | < 0.001 |
| Tuesday 11am – 7pm | 36.328 | 6.177 | < 0.001 |
| Tuesday 7pm – 11pm | -8.426 | 1.576 | < 0.001 |
| Tuesday 11pm – 7am (next day) | -17.567 | 1.641 | < 0.001 |
| Wednesday 7am – 11am | -9.330 | 1.544 | < 0.001 |
| Wednesday 11am – 7pm | 35.424 | 6.163 | < 0.001 |
| Wednesday 7pm – 11pm | -7.876 | 1.564 | < 0.001 |
| Wednesday 11pm – 7am (next day) | -16.180 | 1.637 | < 0.001 |
| Thursday 7am – 11am | -9.780 | 1.545 | < 0.001 |
| Thursday 11am – 7pm | 30.685 | 6.173 | < 0.001 |
| Thursday 7pm – 11pm | -8.398 | 1.566 | < 0.001 |
| Thursday 11pm – 7am (next day) | -15.701 | 1.636 | < 0.001 |
| Friday 7am – 11am | -12.646 | 1.542 | < 0.001 |
| Friday 11am – 7pm | 31.441 | 6.178 | < 0.001 |
| Friday 7pm – 11pm | -8.405 | 1.563 | < 0.001 |
| Friday 11pm – 7am (next day) | -12.757 | 1.633 | < 0.001 |
| Saturday 7am – 11am | -17.945 | 1.548 | < 0.001 |
| Saturday 11am – 7pm | 19.703 | 6.175 | 0.001 |
| Saturday 7pm – 11pm | -11.560 | 1.569 | < 0.001 |
| Saturday 11pm – 7am (next day) | -10.193 | 1.637 | < 0.001 |
| Sunday 7am – 11am | -16.459 | 1.548 | < 0.001 |
| Sunday 11am – 7pm | 22.216 | 6.174 | < 0.001 |
| Sunday 7pm – 11pm | -12.409 | 1.569 | < 0.001 |
| Sunday 11pm – 7am (next day) | -13.832 | 1.636 | < 0.001 |
| Holiday | -8.821 | 1.422 | < 0.001 |
| Holiday +1 day | 3.644 | 1.426 | 0.011 |
| 21-day moving average of arrivals during same-type shifts (lag 5) | 0.389 | 0.086 | < 0.001 |
| Is raining | -0.503 | 0.427 | 0.239 |
| Max temperature exceeds 30$℃$ | -0.428 | 0.612 | 0.484 |

**Supplementary Table 8. Surge-Stage Prediction Model (Missing 5 Days of Input)**

| Covariate | Coefficient | Standard error | P-value |
| --- | --- | --- | --- |
| Intercept | 36.992 | 3.730 | < 0.001 |
| Monday 11am – 7pm | 49.442 | 6.154 | < 0.001 |
| Monday 7pm – 11pm | -5.224 | 1.563 | 0.001 |
| Monday 11pm – 7am (next day) | -15.382 | 1.632 | < 0.001 |
| Tuesday 7am – 11am | -9.242 | 1.554 | < 0.001 |
| Tuesday 11am – 7pm | 36.605 | 6.165 | < 0.001 |
| Tuesday 7pm – 11pm | -8.405 | 1.576 | < 0.001 |
| Tuesday 11pm – 7am (next day) | -17.581 | 1.642 | < 0.001 |
| Wednesday 7am – 11am | -9.327 | 1.545 | < 0.001 |
| Wednesday 11am – 7pm | 35.714 | 6.147 | < 0.001 |
| Wednesday 7pm – 11pm | -7.852 | 1.564 | < 0.001 |
| Wednesday 11pm – 7am (next day) | -16.210 | 1.636 | < 0.001 |
| Thursday 7am – 11am | -9.784 | 1.545 | < 0.001 |
| Thursday 11am – 7pm | 30.991 | 6.154 | < 0.001 |
| Thursday 7pm – 11pm | -8.383 | 1.565 | < 0.001 |
| Thursday 11pm – 7am (next day) | -15.722 | 1.636 | < 0.001 |
| Friday 7am – 11am | -12.633 | 1.542 | < 0.001 |
| Friday 11am – 7pm | 31.731 | 6.162 | < 0.001 |
| Friday 7pm – 11pm | -8.375 | 1.563 | < 0.001 |
| Friday 11pm – 7am (next day) | -12.777 | 1.633 | < 0.001 |
| Saturday 7am – 11am | -17.928 | 1.548 | < 0.001 |
| Saturday 11am – 7pm | 19.990 | 6.160 | 0.001 |
| Saturday 7pm – 11pm | -11.540 | 1.569 | < 0.001 |
| Saturday 11pm – 7am (next day) | -10.214 | 1.637 | < 0.001 |
| Sunday 7am – 11am | -16.452 | 1.548 | < 0.001 |
| Sunday 11am – 7pm | 22.487 | 6.162 | < 0.001 |
| Sunday 7pm – 11pm | -12.389 | 1.569 | < 0.001 |
| Sunday 11pm – 7am (next day) | -13.851 | 1.637 | < 0.001 |
| Holiday | -8.745 | 1.422 | < 0.001 |
| Holiday +1 day | 3.615 | 1.427 | 0.011 |
| 21-day moving average of arrivals during same-type shifts (lag 6) | 0.385 | 0.086 | < 0.001 |
| Is raining | -0.525 | 0.427 | 0.219 |
| Max temperature exceeds 30$℃$ | -0.443 | 0.613 | 0.470 |

**Supplementary Table 9. Surge-Stage Prediction Model (Missing 6 Days of Input)**

| Covariate | Coefficient | Standard error | P-value |
| --- | --- | --- | --- |
| Intercept | 38.448 | 3.725 | < 0.001 |
| Monday 11am – 7pm | 51.859 | 6.151 | < 0.001 |
| Monday 7pm – 11pm | -5.119 | 1.564 | 0.001 |
| Monday 11pm – 7am (next day) | -15.602 | 1.633 | < 0.001 |
| Tuesday 7am – 11am | -9.243 | 1.555 | < 0.001 |
| Tuesday 11am – 7pm | 39.038 | 6.158 | < 0.001 |
| Tuesday 7pm – 11pm | -8.300 | 1.577 | < 0.001 |
| Tuesday 11pm – 7am (next day) | -17.796 | 1.643 | < 0.001 |
| Wednesday 7am – 11am | -9.323 | 1.546 | < 0.001 |
| Wednesday 11am – 7pm | 38.123 | 6.145 | < 0.001 |
| Wednesday 7pm – 11pm | -7.756 | 1.566 | < 0.001 |
| Wednesday 11pm – 7am (next day) | -16.433 | 1.637 | < 0.001 |
| Thursday 7am – 11am | -9.784 | 1.547 | < 0.001 |
| Thursday 11am – 7pm | 33.426 | 6.146 | < 0.001 |
| Thursday 7pm – 11pm | -8.273 | 1.567 | < 0.001 |
| Thursday 11pm – 7am (next day) | -15.950 | 1.636 | < 0.001 |
| Friday 7am – 11am | -12.645 | 1.544 | < 0.001 |
| Friday 11am – 7pm | 34.174 | 6.154 | < 0.001 |
| Friday 7pm – 11pm | -8.271 | 1.564 | < 0.001 |
| Friday 11pm – 7am (next day) | -12.997 | 1.634 | < 0.001 |
| Saturday 7am – 11am | -17.918 | 1.549 | < 0.001 |
| Saturday 11am – 7pm | 22.430 | 6.152 | < 0.001 |
| Saturday 7pm – 11pm | -11.418 | 1.570 | < 0.001 |
| Saturday 11pm – 7am (next day) | -10.427 | 1.638 | < 0.001 |
| Sunday 7am – 11am | -16.438 | 1.549 | < 0.001 |
| Sunday 11am – 7pm | 24.924 | 6.155 | < 0.001 |
| Sunday 7pm – 11pm | -12.278 | 1.570 | < 0.001 |
| Sunday 11pm – 7am (next day) | -14.067 | 1.638 | < 0.001 |
| Holiday | -8.687 | 1.423 | < 0.001 |
| Holiday +1 day | 3.706 | 1.428 | 0.010 |
| 21-day moving average of arrivals during same-type shifts (lag 7) | 0.350 | 0.086 | < 0.001 |
| Is raining | -0.542 | 0.427 | 0.204 |
| Max temperature exceeds 30$℃$ | -0.423 | 0.614 | 0.491 |

**Supplementary Table 10. Prediction Accuracy of the Alternative Surge-Stage Prediction Models**

| Alternative surge-stage prediction models | All data set adj. R^2 | Training set RMSE | Training set MAPE | Test set RMSE | Test set MAPE |
| --- | --- | --- | --- | --- | --- |
| Lag 2 | 0.930 | 8.617 | 12.96% | 8.364 | 13.48% |
| Lag 3 | 0.930 | 8.613 | 12.97% | 8.395 | 13.47% |
| Lag 4 | 0.930 | 8.607 | 12.96% | 8.410 | 13.51% |
| Lag 5 | 0.930 | 8.613 | 12.97% | 8.415 | 13.51% |
| Lag 6 | 0.930 | 8.611 | 12.95% | 8.428 | 13.50% |
| Lag 7 | 0.929 | 8.621 | 12.95% | 8.433 | 13.52% |

**Supplementary Note 2. Supplementary Tables And Figures**

**Supplementary Table 11. Linear Regression Model for Staffing Efficiency (4-Hour Window)**

| Covariate | Coefficient | Standard error | P-value |
| --- | --- | --- | --- |
| Intercept | 560.116 | 46.081 | < 0.001 |
| Monday 7am – 7pm | -124.917 | 33.258 | < 0.001 |
| Monday 7pm – 7am (next day) | -90.090 | 29.637 | 0.002 |
| Tuesday 7am – 7pm | -95.629 | 33.955 | 0.005 |
| Tuesday 7pm – 7am (next day) | -23.325 | 30.505 | 0.445 |
| Wednesday 7am – 7pm | -80.742 | 34.408 | 0.019 |
| Wednesday 7pm – 7am (next day) | -92.692 | 31.816 | 0.004 |
| Thursday 7am – 7pm | -104.434 | 33.586 | 0.002 |
| Thursday 7pm – 7am (next day) | 113.097 | 28.708 | < 0.001 |
| Friday 7pm – 7am (next day) | 29.155 | 32.115 | 0.364 |
| Saturday 7am – 7pm | 26.136 | 28.759 | 0.364 |
| Saturday 7pm – 7am (next day) | -11.548 | 32.957 | 0.726 |
| Sunday 7am – 7pm | 86.724 | 28.780 | 0.003 |
| Sunday 7pm – 7am (next day) | -22.764 | 32.605 | 0.485 |
| Average arrival count per hour | 35.874 | 1.937 | < 0.001 |
| Number of waiting patients at the beginning of the period | 3.640 | 1.522 | 0.017 |
| Number of patients in treatment at the beginning of the period | 5.238 | 0.519 | < 0.001 |
| Number of boarding patients at the beginning of the period | 0.133 | 0.572 | 0.816 |
| Average patient waiting time (minutes) | -3.046 | 0.505 | < 0.001 |
| Average patient LOS (minutes) | 0.009 | 0.040 | 0.825 |
| LWBS rate (%) | -12.856 | 4.676 | 0.006 |
| Experiment stage indicator | -185.657 | 11.151 | < 0.001 |

**Supplementary Table 12. Linear Regression Model for Staffing Efficiency (2-Hour Window)**

| Covariate | Coefficient | Standard error | P-value |
| --- | --- | --- | --- |
| Intercept | 651.598 | 32.285 | < 0.001 |
| Monday 7am – 7pm | -197.699 | 23.597 | < 0.001 |
| Monday 7pm – 7am (next day) | -88.156 | 21.918 | < 0.001 |
| Tuesday 7am – 7pm | -172.423 | 23.671 | < 0.001 |
| Tuesday 7pm – 7am (next day) | -38.886 | 22.731 | 0.087 |
| Wednesday 7am – 7pm | -150.707 | 24.580 | < 0.001 |
| Wednesday 7pm – 7am (next day) | -110.177 | 23.491 | < 0.001 |
| Thursday 7am – 7pm | -171.449 | 23.942 | < 0.001 |
| Thursday 7pm – 7am (next day) | 122.220 | 21.413 | < 0.001 |
| Friday 7pm – 7am (next day) | -29.715 | 22.849 | 0.194 |
| Saturday 7am – 7pm | 33.787 | 21.451 | 0.115 |
| Saturday 7pm – 7am (next day) | -79.034 | 23.208 | < 0.001 |
| Sunday 7am – 7pm | 95.399 | 21.483 | < 0.001 |
| Sunday 7pm – 7am (next day) | -87.039 | 23.016 | < 0.001 |
| Average arrival count per hour | 22.745 | 1.162 | < 0.001 |
| Number of waiting patients at the beginning of the period | 4.083 | 1.185 | < 0.001 |
| Number of patients in treatment at the beginning of the period | 6.681 | 0.365 | < 0.001 |
| Number of boarding patients at the beginning of the period | -0.385 | 0.423 | 0.364 |
| Average patient waiting time (minutes) | -2.216 | 0.314 | < 0.001 |
| Average patient LOS (minutes) | 0.010 | 0.021 | 0.625 |
| LWBS rate (%) | -8.770 | 2.588 | < 0.001 |
| Experiment stage indicator | -189.702 | 8.302 | < 0.001 |

**Supplementary Table 13. Linear Regression Model for Staffing Efficiency (1-Hour Window)**

| Covariate | Coefficient | Standard error | P-value |
| --- | --- | --- | --- |
| Intercept | 703.923 | 23.109 | < 0.001 |
| Monday 7am – 7pm | -242.182 | 16.943 | < 0.001 |
| Monday 7pm – 7am (next day) | -86.974 | 16.037 | < 0.001 |
| Tuesday 7am – 7pm | -222.558 | 16.845 | < 0.001 |
| Tuesday 7pm – 7am (next day) | -45.612 | 16.666 | 0.006 |
| Wednesday 7am – 7pm | -190.612 | 17.716 | < 0.001 |
| Wednesday 7pm – 7am (next day) | -116.126 | 17.167 | < 0.001 |
| Thursday 7am – 7pm | -208.263 | 17.247 | < 0.001 |
| Thursday 7pm – 7am (next day) | 125.558 | 15.705 | < 0.001 |
| Friday 7pm – 7am (next day) | -63.688 | 16.495 | < 0.001 |
| Saturday 7am – 7pm | 37.210 | 15.723 | 0.018 |
| Saturday 7pm – 7am (next day) | -121.772 | 16.616 | < 0.001 |
| Sunday 7am – 7pm | 98.624 | 15.770 | < 0.001 |
| Sunday 7pm – 7am (next day) | -124.292 | 16.552 | < 0.001 |
| Average arrival count per hour | 14.661 | 0.743 | < 0.001 |
| Number of waiting patients at the beginning of the period | 3.698 | 0.896 | < 0.001 |
| Number of patients in treatment at the beginning of the period | 7.455 | 0.264 | < 0.001 |
| Number of boarding patients at the beginning of the period | -0.502 | 0.309 | 0.105 |
| Average patient waiting time (minutes) | -1.385 | 0.183 | < 0.001 |
| Average patient LOS (minutes) | 0.000 | 0.011 | 0.969 |
| LWBS rate (%) | -5.284 | 1.413 | < 0.001 |
| Experiment stage indicator | -193.651 | 6.105 | < 0.001 |

**References**

1. National Centers for Environmental Information. Global Historical Climatology Network

(GHCN)-Daily Dataset. 2022. Available Online: https://www.ncei.noaa.gov/cdo-web (accessed on March 10, 2022).

1. [Hu Y, Cato KD, Chan CW, et al. Use of Real-Time Information to Predict Future Arrivals in the Emergency Department. *Ann Emerg Med*. 2023;81(6):728-737. doi:10.1016/j.annemergmed.2022.11.005](https://www.zotero.org/google-docs/?GCmSfx)
2. [Hu Y, Chan CW, Dong J. Prediction-Driven Surge Planning with Application in the Emergency Department. *Manag Sci*. Published online 2023.](https://www.zotero.org/google-docs/?GCmSfx)
